# HOME HEALTH AIDE AND SOCIAL WORK VISITS IN MEDICARE ADVANTAGE AND TRADITIONAL MEDICARE BY RURALITY

**DOI:** 10.1093/geroni/igae098.1050

**Published:** 2024-12-31

**Authors:** Tracy Mroz, Lisa Garberson, C Holly Andrilla, Davis Patterson

**Affiliations:** University of Washington, Seattle, Washington, United States; University of Washington, Seattle, Washington, United States; University of Washington, Seattle, Washington, United States; University of Washington, Seattle, Washington, United States

## Abstract

Medicare Advantage (MA) enrollment has grown rapidly over the last decade. Prior research suggests MA beneficiaries utilize home health less often than Traditional Medicare (TM) beneficiaries, but research comparing specific services within home health has been limited to individual plans or providers and has not examined rural-urban differences. Home health aide and social work services may be especially important for successful community discharge. Using 2018 100% Medicare files, we conducted hierarchical logistic regression analyses to examine receipt of home health aide and social work visits by MA enrollment and rural-urban status (categorized by Rural Urban Commuting Areas Codes into urban, large rural, small rural, and isolated small rural communities), controlling for beneficiary and community characteristics. Of the 3,143,742 beneficiaries included in analysis, 23% were enrolled in MA versus 77% in TM, and 83% lived in urban communities versus 17% in rural communities. MA versus TM beneficiaries had significantly lower odds of receiving aide and social work visits (OR(95%CI): 0.87(0.82,0.92) and 0.91(0.85,0.98), respectively). Beneficiaries in rural versus urban communities had significantly lower odds of receiving social work visits, with the lowest odds in small rural and isolated small rural communities. However, odds of receiving aide visits were significantly higher in rural versus urban communities and increased as rurality increased. MA beneficiaries in small rural and isolated small rural communities are at particular risk for poor access to social work services, but decreased access to home health aide services for MA beneficiaries may be attenuated for those living in rural communities.

